# Prevalence, Phylogenetic Distribution, Antimicrobial Resistance, and Genetic Relatedness of Extraintestinal Pathogenic *E. coli* (ExPEC) Strains Isolated from Beef Cattle and Slaughterhouse Environment

**DOI:** 10.3390/vetsci12100944

**Published:** 2025-09-30

**Authors:** Resat Ciftci, Husnu Sahan Guran

**Affiliations:** Department of Food Hygiene and Technology, Faculty of Veterinary Medicine, University of Dicle, 21280 Diyarbakır, Turkey; ciftciresat@gmail.com

**Keywords:** extraintestinal pathogenic *Escherichia coli*, cattle, slaughterhouse, antimicrobial susceptibility, clonal diversity, one health

## Abstract

*Escherichia coli* is a commensal bacterium found in the gastrointestinal tracts of animals, and they might serve as a vehicle for transmitting extraintestinal pathogenic *Escherichia coli* (ExPEC) to the environment and human. This study aimed to investigate the prevalence, phylogenetic distribution, antimicrobial resistance, and genetic relatedness of ExPEC in beef cattle, slaughterhouse environment, and workers involved in the evisceration process during slaughter. A total of 447 samples were collected from two slaughterhouses located in the Diyarbakir and Batman cities of the Southeast Anatolia Region of Turkiye. The samples included swabs from beef carcasses (*n* = 133), rectal samples (*n* = 133), hides (*n* = 133), knives (*n* = 18), holding pens (*n* = 12), water (*n* = 6), and workers’ hands (*n* = 12). The samples were analyzed for the presence of ExPEC, and then isolates were screened for antimicrobial susceptibility, phylogenetic groups, and genetic relatedness. A total of 36 out of 447 samples were confirmed to be ExPEC-positive, yielding a prevalence rate of 8.80%. The contamination rates for carcasses, hides, rectal, knives, holding pens, water, and workers’ hands were 10.5%, 6.06%, 6.5%, 5.5%, 8.3%, 0%, and 25%, respectively. The ExPEC isolates were classified into phylogroups D (38.8%), B1 (25.0%), B2 (25.0%), and A (5.5%). Of the isolates, 55.55% (20/36) exhibited resistance to at least one antimicrobial agent from three or more, with the highest resistance observed against ampicillin (61.1%). ERIC-PCR analysis revealed that 35 of the isolates formed 19 distinct clusters, indicating a high level of genetic diversity among the ExPEC isolates. The results of this study demonstrate that beef cattle, the slaughterhouse environment, and workers’ hands can serve as sources of ExPEC contamination.

## Simple Summary

This study examined the presence, genetic characteristics, and antibiotic resistance of extraintestinal pathogenic *Escherichia coli* (ExPEC) in cattle and slaughterhouse environments in southeastern Turkey. ExPEC was detected in 8% of samples, including carcasses, hides, rectal swabs, and particularly workers’ hands (25%), indicating multiple potential points of cross-contamination. The isolates harbored key virulence genes such as *iutA*, *papA* and *papC*, and most belonged to phylogenetic groups B2 and D, which are commonly associated with human infections. High levels of antibiotic resistance were observed, especially against ampicillin and ciprofloxacin, raising significant public health concerns. Genetic analyses revealed diverse yet closely related strains across different sources, further supporting evidence of cross-contamination within the slaughterhouse. Overall, the findings suggest that cattle and slaughterhouse environments may act as reservoirs for antibiotic-resistant ExPEC, underscoring the importance of continuous monitoring and control strategies within a One Health framework.

## 1. Introduction

*Escherichia coli* (*E. coli*) is a Gram-negative, rod-shaped coliform bacterium found as part of the normal microbiota in the gastrointestinal system of both humans and animals [1]. Some pathogenic *E. coli* strains cause intestinal infections (intestinal pathogenic *E. coli*), whereas others cause extraintestinal infections [extraintestinal pathogenic *E. coli* (ExPEC)] [2]. Based on their virulence characteristics, intestinal pathogenic *E. coli* are categorized under the following six pathotypes: enterotoxigenic *E. coli* (ETEC), enteropathogenic *E. coli* (EPEC), enterohemorrhagic *E. coli* (EHEC), enteroaggregative *E. coli* (EAEC), diffusely adherent *E. coli* (DAEC), and enteroinvasive *E. coli* (EIEC). On the other hand, the categorization of extraintestinal pathogenic *E. coli* (ExPEC) according to their virulence characteristics is as follows: uropathogenic *E. coli* (UPEC), neonatal meningitis-associated *E. coli* (NMEC), sepsis-associated *E. coli* (SePEC), avian pathogenic *E. coli* (APEC), mammary pathogenic *E. coli* (MPEC) and endometrial pathogenic *E. coli* (EnPEC) [3,4]. The main distinctive features that differentiate ExPEC from commensal and enteric *E. coli* are their virulence characteristics, which enable them to successfully colonize and infect the host. Among the various virulence factors, the adhesins encoded by the *sfa*, *traT*, *ibeA papA*, *papC*, *iutA*, *kpsMTII*, and *fimH* genes and the HlyA and CNF1 toxins are used to differentiate ExPEC from other *E. coli* [5]. ExPEC strains are predominantly classified in phylogenetic group B2, while a smaller proportion belongs to group D [6].

In molecular epidemiological study on ExPEC, it has been reported that humans, animals, food of animal origin, and environmental sources may serve as potential reservoirs for these pathogens [7,8,9,10,11]. Previous investigations on the presence of ExPEC in animals are observed to have mainly focused on pigs, dairy cattle and chickens [12,13,14]. The prevalence of ExPEC has been reported to range between 5.6 and 10% in pigs, 5.1–29% in dairy cattle, and 10–25% in chickens [10,13,15,16,17,18]. There are only very few studies conducted in slaughterhouse environments in Turkiye on the prevalence and distribution of ExPEC in cattle, and previous studies are observed to have been conducted primarily in the United States of America (USA) and secondly in some European Union (EU) countries [19,20,21].

In a 2014 report by the World Health Organization (WHO), *E. coli* was listed among the top nine microorganisms of international concern, responsible for widespread infections in the community, in hospitals, and through the food chain [22]. Moreover, it is included on the WHO global priority list of critical pathogens for research, discovery, and the development of new antibiotics [23]. Cephalosporins, fluoroquinolones, and trimethoprim-sulfamethoxazole are commonly used to treat *E. coli* infections in both community and hospital settings; however, resistance to these agents has been associated with delays in appropriate therapy, ultimately contributing to increased morbidity and mortality [24]. Reports indicate that ExPEC isolates are generally highly resistant to these first-line antibiotics [25,26,27]. Addressing this challenge requires a One Health approach, recognizing the critical interface between humans, animals, and the environment, since food-producing animals and environmental reservoirs play a significant role in the emergence and dissemination of antimicrobial resistance [11]. In this context, determining the presence of antibiotic-resistant ExPEC in beef cattle and slaughterhouse environments, which constitute a critical node in the food chain, is essential not only for demonstrating the persistence of this pathogen in such settings but also for understanding transmission pathways of ExPEC. To the authors’ knowledge, there is no previous study from Turkiye that has investigated the presence of ExPEC in both cattle and the slaughterhouse environment. This study investigates the presence of ExPEC in beef cattle (hide, rectal, and carcass samples), the slaughterhouse environment, and workers involved in evisceration. Additionally, the phylogenetic background, antimicrobial resistance patterns, and genetic relatedness of the recovered isolates are analyzed.

## 2. Materials and Methods

### 2.1. Study Design and Sample Collection

A cross-sectional study was conducted from March 2022 to September 2022 at two slaughterhouses located in the Diyarbakır and Batman cities of the Southeastern Anatolia region, Turkiye. Two slaughterhouses located in these cities agreed to participate in the study. Both slaughterhouses were small in processing capacity and slaughtered average 30–40 cattle per day. On each sampling day, the number of beef cattle was determined according to the number of animals deemed suitable for slaughter by the official veterinarian within the framework of the legislation in Turkey. Each slaughterhouse was visited three times with a time interval of 4–6 weeks and sampled once per visit. The processing of each animal was followed from start to finish, and samples were collected in the following order: holding pens (*n* = 12), rectal swabs (*n* = 133), hide swabs (*n* = 133), water samples (*n* = 6), hands of slaughterhouse workers (*n* = 12) who were involved in evisceration during slaughtering process, knife swabs (*n* = 8), and carcass swabs (*n* = 133) (Table 1).

### 2.2. Sampling Procedure

The rectal swabs were collected by moistening a cotton swab with sterile 0.1% peptone water. The swab was then inserted 2.5–3.8 cm into the rectum and rotated gently. Hide sample was collected from each animal by swabbing a 1000 cm^2^ area located at the lower abdomen and thoracic regions with a sterile sponge swab (World Bioproducts, Woodinville, WA, USA) [28]. Each carcass surface was sampled using sponge swabs (World Bioproducts, USA) from four sites (rump, flank, brisket, and neck), with each site covering an area of approximately 100 cm^2^ before it entered into the chilling room [29]. Each knife sample was taken with sponge swabs (World Bioproducts, USA) vertically, horizontally and diagonally across the blade and handle [30]. The entire surfaces of both hands of the slaughterhouse personnel involved in the evisceration were sampled with sponge swabs (World Bioproducts, USA) [31]. Holding pen samples were taken with sponge swabs (World Bioproducts, Woodinville, WA, USA) from a representative surface area of the floor of the pre-slaughter resting area of the animals [28]. Samples were collected under aseptic conditions from the slaughterhouse tap water, which originated from the municipal network into 250 mL sterile bottles. All the samples were transported under cooled conditions to the laboratory and analyzed immediately upon arrival.

### 2.3. Isolation of ExPEC in the Samples

*E. coli* isolation from the carcass, hide, rectum, holding pen, knife and hand samples was performed with modifications according to Elsharawy et al. (2022) [31]. First, 40 mL of double-strength MacConkey broth (Neogen, London, UK) was added to each sample and incubated at 37 °C for 18–24 h. After incubation, a loopful of the MacConkey broth culture was subcultured onto MacConkey agar (Neogen, UK) and incubated at 37 °C for 18–24 h. Up to three presumptive *E. coli* colonies were selected and streaked onto Eosin-Methylene Blue (EMB) agar (Merck, Darmstadt, Germany) and incubated at 37 °C for 18–24 h. Colonies exhibiting a reflective metallic green color on EMB agar were further analyzed using standard biochemical test (indole). To isolate *E. coli* from water samples, 10 mL portions were taken from each sample and transferred into 100 mL sterile bottles, followed by the addition of 40 mL of double-strength MacConkey broth. The samples were incubated at 37 °C for 18–24 h and subsequently analyzed using the same procedure as described for the other samples.

### 2.4. Detection of Virulence Genes Associated with ExPEC

DNA extraction from presumptive *E. coli* isolates was carried out using the boiling method, as described by Queipo-Ortúno et al. (2008) [32]. The PCR-based confirmation was performed for the isolates identified as *E. coli* with the conventional culture technique, with the method described by Wang et al. (1996) [33], and it involved the amplification of the 16S rRNA gene region. The PCR mixture was prepared in 25 µL reaction volumes containing 2.5 μL 10× PCR buffer, 2.5 μL MgCl_2_, 2 μL dNTPs, 0.2 μL Taq DNA polymerase (5 U/µL), 0.9 μL of each primer (20 pmol) (Table 2), 11 μL sterile water and 5 μL template DNA. The PCR program consisted of an initial denaturation at 95 °C for 5 min followed by 35 cycles of denaturation at 95 °C for 1 min, annealing at 52 °C for 1 min, extension at 72 °C for 1 min, and 1 cycle of a final extension at 72 °C for 7 min. The isolates confirmed as *E. coli* by PCR were considered ExPEC based on the presence of the *papA*, *papC*, *iutA*, *kpsMTII* and *fimH* virulence genes. ExPEC isolates were confirmed using a modified multiplex PCR protocol (Johnson et al., 2003; Zhu et al., 2017) [12,34], targeting virulence genes with amplicon sizes ranging from 203 to 717 bp. The PCR mixture was prepared in a reaction volume of 50 µL containing 6 μL 10× PCR buffer, 8 μL MgCl_2_, 8 μL dNTPs, 0.6 μL Taq DNA polymerase (5 U/µL), 1 μL of each 40 pmol primer (Table 2), 12 μL sterile water and 5.4 μL template DNA. The PCR amplification was performed as follows: an initial denaturation at 95 °C for 5 min followed by a total of 35 PCR cycles of denaturation at 95 °C for 1 min, annealing at 52 °C for 1 min, extension at 72 °C for 1 min, and 1 cycle of a final extension of 7 min at 72 °C.

### 2.5. Antimicrobial Susceptibility Testing

The antibiotic susceptibility of the ExPEC isolates was determined using a BD Phoenix™ M50 device (BD Diagnostic Instrument Systems, Sparks, MD, USA) according to the manufacturer’s instructions. For this purpose, BD Phoenix™ NMIC/ID-433 panel cards containing the antibiotics amikacin, amoxicillin-clavulanate, ampicillin, ampicillin-sulbactam, cefazolin, cefepime, ceftazidime, ceftolozane-tazobactam, ceftriaxone, cefuroxime, ciprofloxacin, ertapenem, gentamicin, imipenem, levofloxacin, meropenem, piperacillin-tazobactam, tigecycline and trimethoprim-sulfamethoxazole, were used. Following analyses, the minimum inhibitory concentrations (MICs) were assessed according to the recommendations of the European Committee on Antimicrobial Susceptibility Testing (EUCAST, 2023). Isolates exhibiting resistance to at least one antimicrobial agent from three or more antimicrobial categories were classified as multidrug-resistant [37].

### 2.6. Phylogenetic Group Determination

The phylogenetic groups of confirmed ExPEC isolates were determined by PCR as reported by Clermont et al. (2000) [35]. Isolates were assigned to one of four groups (A, B1, B2, or D) based on their possession of two genes (*chuA* and *yjaA*) and a TSPE4.C2 genetic marker. The PCR mixture was prepared in a reaction volume of 50 µL and contained 6 μL 10× PCR buffer, 8 μL MgCl_2_, 8 μL dNTPs, 0.5 μL Taq DNA polymerase (5 U/µL), 1 μL of each 40 pmol primer (Table 2), 16.5 μL sterile water and 5 μL template DNA. The PCR amplification program was as follows: an initial denaturation at 94 °C for 5 min followed by a total of 35 PCR cycles of denaturation at 94 °C for 30 s, hybridization at 55 °C for 30 s, and extension at 72 °C for 30 s with 1 cycle of a final extension of 72 °C for 7 min.

### 2.7. DNA Fingerprinting and Phylogenetic Analysis

The clonal variability and phylogenetic closeness among the ExPEC isolates were determined using the enterobacterial repetitive intergenic consensus-polymerase chain reaction (ERIC-PCR) method described by Versalovic et al. (1991) [36]. The primers used for this purpose are presented in Table 2. The PCR mixture was carried out in 25 μL of the DreamTaq Hot Start Green PCR Master Mix (Thermo Fisher, Waltham, MA, USA) and the primers. The initial template denaturation step consisted of 94 °C for 1 min, followed by 40 cycles of 94 °C for 1 min, annealing at 25 °C for 1 min, and extension at 72 °C for 2 min. The PCR products were run on 2% agarose gel using a current of 100 volts by electrophoresis. The resulting bands were imaged using the Image Lab Software v6.0 gel documentation system for analysis.

### 2.8. Statistical Analyses

The statistical significance of the differences observed for the presence of ExPEC, sample type (carcass, hide and rectum) and the city from which the samples were collected (Diyarbakir and Batman) was analyzed with Pearson’s chi-squared (χ^2^) test. Descriptive statistics are presented in percentages. The confidence intervals (two-sided 95% CI [lower limit, upper limit]) of proportions was calculated. The statistical analyses were performed using the SPSS (version 24) software package. *p* < 0.05 was considered significant.

The cluster analysis of the agarose gel band models of the ERIC-PCR products was performed using an unweighted pair group method with arithmetic mean (UPGMA) [38]. The phylogenetic trees were constructed with iTOL version 4 [39]. After normalization, profile similarities were calculated using the Jaccard similarity coefficient based on peak patterns.

## 3. Results

### 3.1. Prevalence of ExPEC

*E. coli* was identified in 409 of the 447 samples analyzed, representing 91.49% of the total samples. All *E. coli* isolates confirmed by PCR were tested for the presence of five ExPEC-associated genes (*papA*, *papC*, *iutA*, *kpsMTII*, and *fimH*) (Table 2). Among the isolates classified as *ExPEC* based on the presence of at least two virulence genes, 38.8% (14/36) were recovered from carcass samples, 22.2% (8/36) from hide samples, and 25% (9/36) from rectal samples. No significant difference was observed in ExPEC distribution among carcass, hide, and rectum samples (*p* > 0.05) (Table 3). Out of the 133 beef cattle, only one tested positive for *ExPEC* in both the carcass and rectal samples. ExPEC was detected in 8.3% (3/36) of worker hand samples, 2.7% (1/36) of knife samples, and 2.7% (1/36) of holding pen samples (Table 3). No significant difference in ExPEC prevalence was observed between Diyarbakır (8.10%, 17/210) and Batman (8.02%, 19/237) (*p* > 0.05).

### 3.2. Virulence Genes Associated with ExPEC

Five virulence factor-encoding genes (*papA*, *papC*, *iutA*, *kpsMTII*, *and fimH*) were employed for the molecular detection of ExPEC (Table 2). Among these, the most frequently detected were *fimH* (100%), *iutA* (97.2%), *papC* (16.6%), and *papA* (13.8%), while *kpsMTII* (0.0%) was not detected. Five distinct virulence gene combinations were identified, ranging from two to four genes per isolate. The most common combination was *iutA*/*fimH*, detected in 28 isolates (77.7%), followed by *papA*/*papC*/*iutA*/*fimH* in 3 isolates (8.3%), *papA*/*iutA*/*fimH* in 2 isolates (5.5%), *papC*/*iutA*/*fimH* in 2 isolates (5.5%), and *papC*/*fimH* in 1 isolate (2.7%).

### 3.3. Antimicrobial Resistance

The percentages of resistance of the 36 ExPEC isolates to amoxicillin-clavulanate, ampicillin, ampicillin-sulbactam, cefazolin, cefepime, ceftazidime, ceftriaxone, cefuroxime, ciprofloxacin, gentamicin, levofloxacin, tigecycline and trimethoprim-sulfamethoxazole were ascertained as 27.7% (10/36), 61.1% (22/36), 13.8% (5/36), 16.6% (6/36), 8.3% (3/36), 8.3% (3/36), 16.6% (6/36), 16.6% (6/36), 38.8% (14/36), 22.2% (8/36), 38.8% (14/36), 30.5% (11/36) and 47.2% (17/36), respectively. All of the isolates (100%) were found to be susceptible to amikacin, ceftolozane-tazobactam, ertapenem, imipenem, meropenem and piperacillin-tazobactam. Furthermore, 55.5% of the isolates were multidrug-resistant (Table 4 and Table 5).

### 3.4. Phylogenetic Grouping and DNA Fingerprinting

The ExPEC isolates predominantly belonged to phylogenetic group D (14/36, 38.88%), followed by groups B1 (9/36, 25%) and B2 (9/36, 25%), while a small proportion was classified as group A (2/36, 5.5%). Additionally, 5.5% (2/36) of the isolates could not be assigned to any phylogenetic group.

**Figure 1:**
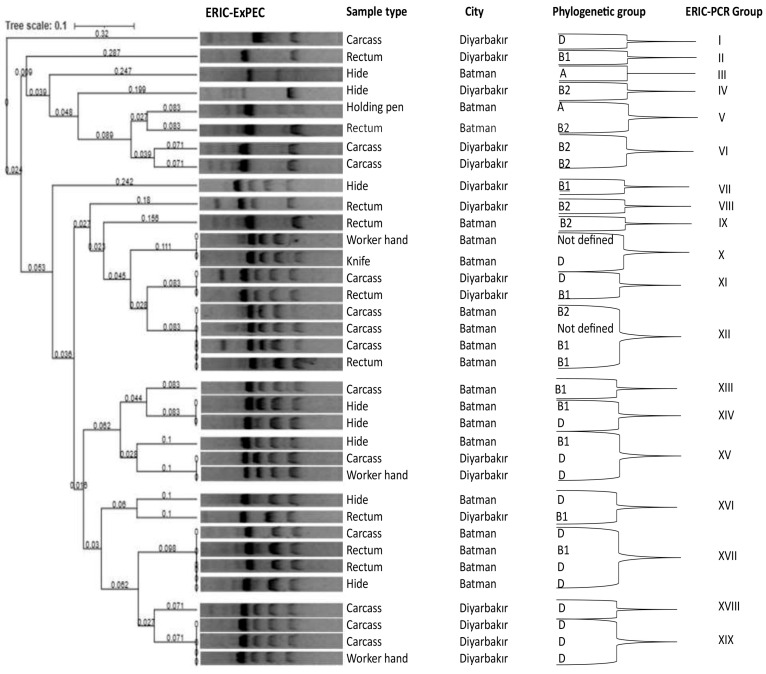
Dendrogram produced by cluster analysis of the ERIC-PCR fingerprinting data (UPGMA) of the ExPEC-positive samples, based on a Dice coefficient. Nineteen major clusters (I to XIX) of related samples were defined. For each ERIC-PCR fingerprint, band sizes, sample type, and locations of the samples are shown.

The rate of similarity between the ExPEC isolates was determined by UPGMA analysis and the rate of similarity was ascertained to be >85%, using the Jaccard similarity coefficient. The number of bands per isolate ranged from 1 to 5, with sizes between 100 and 1500 bp. However, no band was observed for one of the isolates. In total, out of the 35 ExPEC isolates, 26 were classified into 9 clusters, while the remaining 9 isolates were assigned to 9 individual clusters, resulting in a total of 19 different clusters (Figure 1). Clusters XII and XVII were the largest, each comprising 4 isolates, followed by clusters XV and XIX with 3 isolates each. Clusters V, VI, X, XI, XIV, and XVI contained 2 isolates each. A dendrogram analysis of the ERIC-PCR band patterns of the ExPEC isolates is shown in Figure 1.

## 4. Discussion

The contamination of food of animal origin with ExPEC may occur at any stage of the food chain from the farm to the table/fork [11,40,41]. Research conducted in various countries has shown that animal products such as poultry meat and pork may serve as reservoirs for ExPEC pathotypes [7,14,42]. This study addressed *ExPEC* and their antibiotic resistance profiles and genetic relatedness associated with beef cattle and slaughterhouse environment in Turkey. Based on our findings, the overall prevalence in the samples (*n* = 447) was 8%. Although there is limited data on the presence of ExPEC in beef cattle worldwide, several studies have observed its prevalence in poultry and pigs at the slaughterhouse level [10,13,17,18]. In one of the very few studies available on the investigation of the presence of ExPEC in cattle and the slaughterhouse environment, Schmidt et al. (2015) [20], reported that, out of the 184 cattle, 0.36% were ExPEC and these samples originated from the hide samples. Based on these results, the researchers suggested that the hide, carcass and rectum of the cattle were not major reservoirs of ExPEC. Considering that slaughterhouses play a critical role in human–animal–environment interactions, although the overall ExPEC prevalence in our study was modest (8%), its detection on carcasses, hides, rectal swabs, and particularly on workers’ hands (25%) underscores multiple potential cross-contamination points during evisceration and dressing, which may facilitate the transmission of ExPEC strains into the food chain. Literature reports indicate that the presence of ExPEC in the meat and meat products of various animal species sold at the retail level ranges from 5.1% to 25% [14,17,21,43]. Upon testing ground beef, meat grinder and staff hand samples, Santo et al. (2007) [44], reported that, out of the 287 *E. coli* isolates recovered, 3 isolates from ground beef and 2 from the meat grinder were identified as ExPEC. The finding that 25% of workers’ hands were contaminated with ExPEC in this study suggests that, in addition to posing risks to the workers themselves, they may also serve as vectors for the dissemination of ExPEC within the slaughterhouse environment and potentially into the community.

Medium- and large-scale slaughterhouses can process more animals than small ones. This increases the risk of fecal and cross-contamination and makes it more challenging to maintain proper hygiene compared to small-scale facilities. In this context, the prevalence of 8% detected in this study conducted in two small slaughterhouses suggests that in medium- and large-scale facilities, where cattle are sourced from wider areas and animal density is higher, the prevalence of ExPEC, as well as clonal and resistance diversity, may be relatively higher. Nevertheless, it is not possible to draw a definitive conclusion on this issue, since contamination risk is not only determined by the size or capacity of the slaughterhouse [45].

ExPEC show differences from both commensal *E. coli* and shiga-toxigenic *E. coli* with respect to presence of certain virulence factors) [46,47]. The identification of *E. coli* isolates as ExPEC is primarily based on the detection of the presence of the *papA*, *papC*, *iutA* and *kpsMTII* genes, and secondarily based on the presence of the *fimH* gene [15,48]. In the present study, the isolates identified as ExPEC were determined to carry the *papA*, *papC*, *iutA*, and *fimH* genes at rates of 13.8% (5/36), 16.6% (6/36), 97.2% (35/36), and 100% (36/36), respectively. Similarly to our study, there are reports indicating the frequent occurrence of the *fimH* and *iutA* genes in ExPEC isolates [19,20,49]. Xia et al. (2011) [15], determined that, of the ExPEC isolates they recovered from ground beef, 98.5% (197/200) carried the *iutA* gene, 49% (98/200) carried the *papC* gene and 10% (20/200) possessed the *papA* gene. However, in contrast to beef, several studies show that the prevalence of ExPEC-associated virulence genes is higher in chicken meat, followed by pork) [11]. Recent studies on cattle-derived ExPEC have shown a broader virulence repertoire than that evaluated in this study. We focused on the virulence genes *fimH*, *iutA*, *papC*, *papA*, and *kpsMTII*. However, virulence genes such as *iss*, *traT*, *ompT*, *hlyA*, *cnf2*, *vat*, *tsh*, *ibeA*, *cvaC*, *aer*, *sfa/focDE*, *iha*, *iucD*, *bmaE*, *fyuA*, *irp2*, *ireA* and *iroN* have been reported for ExPEC in different studies [50,51,52,53]. In addition, ExPEC strains associated with bovine mastitis have also been found to carry secretion system genes, including T6SS and T4SS, which demonstrates the diversity of virulence genes present in cattle reservoirs [52].

While the majority of *E. coli* strains belong to the phylogenetic group A, most of the ExPEC strains are indicated to belong to the phylogenetic groups B2 and D [35,54]. In this study, the ExPEC isolates were classified into phylogenetic groups A, B1, B2, and D, with frequencies of 5.55% (2/36), 25% (9/36), 25% (9/36), and 38.88% (14/36), respectively. Similarly to our findings, Xia et al. (2011) [15], determined the distribution of the ExPEC isolates recovered from ground beef into the phylogenetic groups A, B1, B2 and D as 10%, 0.0%, 20% and 70%, respectively. On the other hand, different from the present study, Boudjerda and Lahouel (2022) [14], determined the distribution of ExPEC isolates of beef origin between the phylogenetic groups A, B1, B2 and D as 64.3% (47/73), 25.9% (20/73), 3.9% (3/73), and 3.8% (3/73), respectively. Micenková et al. (2016) [47], determined that while the majority of the 407 ExPEC isolates that they recovered from human clinical samples belonged to the phylogenetic group B2 (52.6%, 214/407), the order of distribution of the isolates for the other groups was as follows: group D—18.4% (75/407), group A—18.4% (75/407) and group B1—10.6% (43/407). Chakraborty et al. (2015) [55], determined that, of the 300 ExPEC isolates they recovered from human urinary infection and sepsis cases, 36% (108/300) belonged to the phylogenetic group D, 35% (104/300) to Group B2, 20% (61/300) to Group A and 9% (27/300) to Group B1. In a study by Tayh et al. (2023) [56], it was reported that of 42 ExPEC isolates recovered from human clinical samples, 54.8% (23/42) belonged to the phylogenetic group B2 and 45.2% (19/42) belonged to the phylogenetic group D. Therefore, the phylogenetic distribution of ExPEC isolates in this study was found to be similar to that reported in previous research involving human clinical samples [54].

Although antibiotics are commonly used for treating infections, they have also been inappropriately used at sub-therapeutic levels in animal feed and as growth-promoting agents in livestock production [57]. In recent years, it has been reported that *E. coli*, and, in particular, ExPEC, have gained a significantly increasing resistance to first-line antibiotics [46,58]. The present study demonstrated that the ExPEC isolates showed the highest level of resistance to ampicillin (61.1%, 22/36), and was followed by ciprofloxacin (38.8%, 14/36), and amoxicillin-clavulanate (27.77%, 10/36). Santo et al. (2007) [44], reported to have detected resistance to tetracycline and streptomycin in ExPEC isolates from ground beef and meat grinder swab samples. Upon recovering ExPEC isolates from ground beef samples, Xia et al. (2011) [15], determined that 67% of the strains were resistant to tetracycline, 31.5% to gentamicin, and 22.5% to ampicillin, and indicated that all were susceptible to amikacin and ciprofloxacin. Boudjerda and Lahouel (2022) [14], reported that the ExPEC isolates they recovered from chicken meat, beef and raw milk showed the highest level of resistance to tetracycline (64.5%) followed by amoxicillin (54.04%), ampicillin (53.61%), sulfonamide (43.82%), trimethoprim (37.87%), trimethoprim-sulfamethoxazole (37.44%), and ciprofloxacin (17.87%). Differences between the antibiotic resistance profiles of ExPEC isolates are attributed to factors related to sample type (animal, food, clinic, environment), sample number, region and the use of antibiotics in the treatment of infections [14,43,48,59]. The high prevalence of resistant ExPEC strains in this study poses potential risks, including subclinical colonization of the human intestinal tract through direct contact or consumption of contaminated meat until favorable conditions allow extraintestinal infection, as well as the transfer of resistance genes to the human microbiota [60].

ERIC-PCR has gained widespread use for determining the clonal relatedness of *ExPEC* isolates from clinical, environmental, animal, and food samples, owing to its capacity to provide rapid and reliable results [3,61]. In the present study, the *ExPEC* isolates, classified into 19 distinct clusters, can be attributed to the diverse sources of the samples (carcass, hide, rectum, holding pen, knife, etc.), which were collected on different dates and from two distinct cities. Sarowska et al. (2022) [62], reported a heterogenous distribution for the clonal relatedness of ExPEC isolates recovered from different materials, including chicken feces collected from cages and litter on poultry farms, cloacal swabs and raw meat sold retail (chicken meat, turkey meat, pork, beef). The results of the present study demonstrated that the ExPEC isolates classified into the same cluster showed high similarity to each other. This suggests the possibility of cross-contamination between the carcass, hide and rectal swab samples, as well as the hand and knife samples.

This study provides important data on the antibiotic resistant ExPEC strains isolated from cattle, slaughterhouse environments, and slaughterhouse workers in the cities of Diyarbakır and Batman in southeastern Turkey. However, there were limitations in the present study. Sampling was conducted at two slaughterhouses in Diyarbakır and Batman, located in Southeastern Turkiye. Both slaughterhouses have relatively small processing capacities and may not be representative of slaughterhouses in Turkiye or facilities with different processing scales. Phylogenetic analyses in this study were conducted using the method proposed by Clermont et al. (2000) [35], and two ExPEC isolates could not be classified. This represents a limitation, as more recent methods described in the literature allow the assignment of *E. coli* isolates to a larger number of phylogenetic groups [63]. ERIC-PCR was used to determine genetic relatedness among ExPEC isolates. While this method is cost-effective and provides valuable information about clonal diversity, it has lower discriminatory power than advanced molecular typing methods such as multi-locus sequence typing (MLST) or whole genome sequencing (WGS). In this context, findings regarding possible transmission routes or epidemiological links between sources are indicative.

## 5. Conclusions

In conclusion, the obtained results suggest that cattle and the slaughterhouse environment may act as reservoirs for antibiotic-resistant *ExPEC* strains linked to human infections. To fully understand the transmission dynamics between these sources and *ExPEC*, further in-depth investigations are needed to elucidate transmission pathways and risk factors. Regular and systematic screening of potentially zoonotic ExPEC in clinical, environmental, and animal samples, within the framework of a One Health approach, is crucial. This would not only enhance understanding of the pathogen’s epidemiology but also support the development of effective public health protection and control strategies.

## Figures and Tables

**Table 1:** Number and distribution of samples collected in Diyarbakir and Batman city.

Samples	Diyarbakır	Batman	Number of Samples
Carcass	62	71	133
Hide	62	71	133
Rectum	62	71	133
Knife	9	9	18
Workers’ hand	6	6	12
Holding pen	6	6	12
Water	3	3	6
Total	210	237	447

**Table 2:** Primers used to amplify target genes.

Target Gene	Primer Sequence (5′–3′)	Amplicon Length (bp)	PCR Analysis (Method)	Reference
*E. coli* *16S rRNA*	F: GACCTCGGTTTAGTTCACAGA R: CACACGCTGACGCTGACCA	585	*E.coli* confirmation (simplex PCR)	[33]
*papA*	F: ATGGCAGTGGTGTCTTTTGGTG R: CGTCCCACCATACGTGCTCTTC	717	ExPEC virulence gene analysis (Multiplex PCR)	[12,34]
*papC*	F: GTGGCAGTATGAGTAATGACCGTTA R: ATATCCTTTCTGCAGGGATGCAATA	203		
*iutA*	F: ATCGGCTGGACATCATGGGAAC R: CGCATTTACCGTCGGGAACGG	314		
*kpsMTII*	F: GCGCATTTGCTGATACTGTTG R: CATCCAGAC GATAAGCATGAGCA	272		
*fimH*	F: TGCAGAACGGATAAGCCGTGG R: GCAGTCACCTGCCCTCCGGTA	508		
*chuA*	F: GACGAACCA ACGGTCAGGAT R: TGCCGCCAGTACC AAAGACA	279	Phylogenetic group analysis (Triplex PCR)	[35]
*yjaA*	F: TGAAGTGTCAGGAGACGCT G R: ATGGAGAATGCGTTCCTCAAC	211		
TspE4.C2	F: GAGTAATGTCGGGGCATTCA R: CGCGCCAACAAAGTATTACG	152		
ERIC	ERIC1: ATGTAAGCTCCTGGGGATTCAC ERIC2:AAGTAAGTGACTGGGGTG AGCG	Variable	Genotyping (ERIC PCR)	[36]

**Table 3:** Prevalence of *ExPEC* in slaughterhouses by sample type and city.

Sample Type	No. of Samples	No. (%) of Positive Samples	95% Cl *
Carcass	133	14 (10.53%)	6.37–16.89
Hide	133	8 (6.02%)	3.08–11.42
Rectum	133	9 (6.77%)	3.60–12.36
Knife	18	1 (5.56%)	0.99–25.76
Holding pen	12	1 (8.33%)	1.49–35.39
Workers’ hand	12	3 (25.00%)	8.89–53.23
Water	6	0 (0.00%)	0.00–39.03
**City**			
Diyarbakır	210	17 (8.10%)	5.12–12.58
Batman	237	19 (8.02%)	5.19–12.18
*Overall prevalence*	447	36 (8.00%)	6.00–11.00

* CI = Confidence interval.

**Table 4:** Resistance to antibiotics among ExPEC isolates.

Antibiotic Class	No. (%) Antibiotic-Resistant ExPEC Isolates *							
	Antibiotic Agents	Carcass (*n* = 14)	Hide (*n* = 8)	Rectum (*n* = 9)	Knife (*n* = 1)	Workers Hand (*n* = 3)	Holding Pen (*n* = 1)	Total (*n* = 36)
Beta-Lactams								
Penicilins	Amoxicillin-Clavulate	5 (35.7%)	0 (0%)	3 (33.3%)	0 (0%)	2 (66.7%)	0 (0%)	10 (27.8%)
	Ampicillin	10 (71.4%)	3 (37.5%)	5 (55.6%)	1 (100%)	3 (100%)	0 (0%)	22 (61.1%)
	Ampicilin-Sulbactam	3 (21.4%)	0 (0%)	1 (11.1%)	0 (0%)	1 (33.3%)	0 (0%)	5 (13.9%)
Cephalosporins	Cefazolin	4 (28.6%)	0 (0%)	1 (11.1%)	0 (0%)	1 (33.3%)	0 (0%)	6 (16.7%)
	Cefepime	3 (21.4%)	0 (0%)	0 (0%)	0 (0%)	0 (0%)	0 (0%)	3 (8.3%)
	Ceftazidime	3 (21.4%)	0 (0%)	0 (0%)	0 (0%)	0 (0%)	0 (0%)	3 (8.3%)
	Ceftriaxone	4 (28.6%)	0 (0%)	1 (11.1%)	0 (0%)	1 (33.3%)	0 (0%)	6 (16.7%)
	Cefuroxime	4 (28.6%)	0 (0%)	1 (11.1%)	0 (0%)	1 (33.3%)	0 (0%)	6 (16.7%)
Fluoroquinolones	Ciprofloxacin	8 (57.1%)	2 (25%)	2 (22.2%)	0 (0%)	2 (66.7%)	0 (0%)	14 (38.9%)
	Levofloxacin	8 (57.1%)	2 (25%)	2 (22.2%)	0 (0%)	2 (66.7%)	0 (0%)	14 (38.9%)
Aminoglycosides	Gentamicin	4 (28.6%)	0 (0%)	3 (33.3%)	0 (0%)	1 (33.3%)	0 (0%)	8 (22.2%)
Tetracyclines	Tigecycline	7 (50%)	0 (0%)	2 (22.2%)	1 (100%)	1 (33.3%)	0 (0%)	11 (30.6%)
Sulfonamides	Trimethoprim-Sulfametxazole	10 (71.4%)	3 (37.5%)	0 (0%)	1 (100%)	3 (100%)	0 (0%)	14 (38.9%)

* All isolates were pan-susceptible to Amicasin, Ceftolozane-Tazobactam, Ertapenem, Imipenem, Meropenem and Piperacillin-Tazobactam.

**Table 5:** Virulence factor genes, phylogenetic group and antibiotic-resistant phenotypes among ExPEC isolates obtained from slaughterhouses by sample type in Turkiye.

No	Sample Type	City	ExPEC Virulence Gene *	Phylogeny	Phenotypic Antibiotic Resistance Profile	Multidrug Resistance
1	Carcass	Diyarbakır	*papA*, *iutA*, *fimH*	D	-	−
2	Carcass	Diyarbakır	*papA*, *papC*, *iutA*, *fimH*	D	AMP,CIP,LEV,TGC,TMP-SMX *(5)*	+
3	Carcass	Diyarbakır	*papA*, *papC*, *iutA*, *fimH*	D	AMP,CIP,LEV,TGC,TMP-SMX *(5)*	+
4	Carcass	Diyarbakır	*papA*, *papC*, *iutA*, *fimH*	D	AMP,CIP,LEV,TGC,TMP-SMX *(5)*	+
5	Carcass	Diyarbakır	*papA*, *iutA*, *fimH*	D	AMC,AMP,CIP,GEN,LEV,TGC,TMP-SMX *(7)*	+
6	Carcass	Diyarbakır	*iutA*, *fimH*	B2	AMP,CFZ,FEP,CAZ,CRO,CXM,CIP,GEN,LEV,TMP-SMX *(10)*	+
7	Carcass	Diyarbakır	*iutA*, *fimH*	B2	-	−
8	Carcass	Diyarbakır	*iutA*, *fimH*	D	AMC,AMP,SAM,CFZ,CRO,CXM,CIP,GEN,LEV,TGC,TMP-SMX *(11)*	+
9	Carcass	Diyarbakır	*iutA*, *fimH*	B2	AMP,CFZ,FEP,CAZ,CRO,CXM,CIP,GEN,LEV,TMP-SMX *(10)*	+
10	Hide	Diyarbakır	*iutA*, *fimH*	B1	AMP,CIP,LEV,TMP-SMX *(4)*	+
11	Hide	Diyarbakır	*iutA*, *fimH*	B2	-	−
12	Rectum	Diyarbakır	*iutA*, *fimH*	B2	AMP,CIP,GEN,LEV,TMP-SMX *(5)*	+
13	Rectum	Diyarbakır	*iutA*, *fimH*	B1	-	−
14	Rectum	Diyarbakır	*iutA*, *fimH*	B1	AMC,AMP,GEN *(3)*	+
15	Rectum	Diyarbakır	*iutA*, *fimH*	B1	AMC,AMP,SAM,CFZ,CRO,CXM,CIP,GEN,LEV,TGC *(10)*	+
16	Worker hand	Diyarbakır	*papC*, *iutA*, *fimH*	D	AMP,CIP,LEV,TGC,TMP-SMX *(5)*	+
17	Worker hand	Diyarbakır	*iutA*, *fimH*	D	AMC,AMP,SAM,CFZ,CRO,CXM,CIP,GEN,LEV,TMP-SMX *(10)*	+
18	Carcass	Batman	*iutA*, *fimH*	B1	AMC,AMP,SAM,CFZ,FEP,CAZ,CRO,CXM,CIP,LEV,TMP-SMX *(11)*	+
19	Carcass	Batman	*iutA*, *fimH*	ND	-	−
20	Carcass	Batman	*iutA*, *fimH*	B1	AMC,AMP,SAM,TGC,TMP-SMX *(5)*	+
21	Carcass	Batman	*iutA*, *fimH*	D	TGC	+
22	Carcass	Batman	*iutA*, *fimH*	B2	AMC,AMP,TMP-SMX *(3)*	+
23	Hide	Batman	*iutA*, *fimH*	D	-	−
24	Hide	Batman	*iutA*, *fimH*	A	-	−
25	Hide	Batman	*papC*, *fimH*	D	-	−
26	Hide	Batman	*iutA*, *fimH*	D	-	−
27	Hide	Batman	*iutA*, *fimH*	B1	AMP,TMP-SMX *(2)*	−
28	Hide	Batman	*iutA*, *fimH*	B1	AMP,CIP,LEV,TMP-SMX *(4)*	+
29	Rectum	Batman	*iutA*, *fimH*	D	-	−
30	Rectum	Batman	*iutA*, *fimH*	B1	-	−
31	Rectum	Batman	*papC*, *iutA*, *fimH*	B2	-	−
32	Rectum	Batman	*iutA*, *fimH*	B2	AMP,TMP-SMX *(2)*	−
33	Rectum	Batman	*iutA*, *fimH*	B2	AMC,AMP,TGC *(3)*	+
34	Knife	Batman	*iutA*, *fimH*	D	AMP,TGC,TMP-SMX *(3)*	+
35	Worker hand	Batman	*iutA*, *fimH*	ND	AMC,AMP,TMP-SMX *(3)*	+
36	Holding pen	Batman	*iutA*, *fimH*	A	-	−

* All isolates were negative for the *kpsMTII* virulence gene. Amoxicillin-Clavulate (AMC), Ampicillin (AMP), Ampicilin-Sulbactam (SAM), Cefazolin (CFZ), Cefepime (FEP), Ceftazidime (CAZ), Ceftriaxone (CRO), Cefuroxime (CXM), Ciprofloxacin (CIP), Gentamicin (GEN), Levofloxacin (LEV), Tigecycline (TGC),Trimethoprim-Sulfametxazole (TMP-SMX). Italic numbers in parentheses (e.g., 3) show the number of antibiotics resistant.

## Data Availability

The original contributions presented in this study are included in the article. Further inquiries can be directed to the corresponding author(s).
